# Association of Maternal and Child Nutritional Status in Brazil: A Population Based Cross-Sectional Study

**DOI:** 10.1371/journal.pone.0087486

**Published:** 2014-01-24

**Authors:** Mariana Santos Felisbino-Mendes, Eduardo Villamor, Gustavo Velasquez-Melendez

**Affiliations:** 1 Department of Maternal and Child Nursing and Public Health, Escola de Enfermagem, Universidade Federal de Minas Gerais (UFMG), Belo Horizonte, Minas Gerais, Brazil; 2 Department of Epidemiology, School of Public Health, University of Michigan, Ann Arbor, Michigan, United States of America; University of Washington, United States of America

## Abstract

**Background:**

Although child undernutrition and stunting has been decreasing worldwide while obesity rates increase, these extreme conditions might coexist in families from low- and middle-income countries. We examined the association between maternal and child anthropometric indicators using a population representative sample.

**Methods:**

4,258 non-pregnant women and their children <60 months who participated in the 2006 Brazilian Demographic Health Survey. We compared the distributions of two nutritional indexes of children, height-for-age (HAZ) and body mass index-for age (BAZ) z-scores, by categories of maternal height, body mass index (BMI), and waist circumference (WC). Adjusted mean differences and 95% confidence intervals (95% CI) were estimated from linear regression, taking into account the complex survey design. We also examined the associations of maternal anthropometry with the prevalence of child stunting (HAZ<−2) and overweight/obesity (BAZ>2).

**Results:**

HAZ was positively associated with maternal height and WC in a linear fashion. After adjustment, for sociodemographic characteristics, children whose mothers' height was<145 cm had 1.2 lower HAZ than children whose mothers were ≥160 cm tall (p-trend<0.0001). After further adjustment for maternal height and maternal BMI, children of mothers with a waist circumference ≥88 cm had 0.3 higher HAZ than those of mothers with WC<80 cm (p-trend<0.01). Adjusted prevalence ratios and 95% CI for stunting by the categories of maternal height (<145, 145–149, 150–154, 155–159 and ≥160 cm) were, respectively, 2.95 (1.51;5.77), 2.29 (1.33;3.93), 1.09 (0.63;1.87), and 0.89 (0.45;1.77), (p-trend = 0.001). BAZ was positively associated with maternal BMI and WC.

**Conclusion:**

We observed a strong, positive association of maternal and child nutritional status. Mothers of low stature had children with lower stature, mothers with central obesity had taller children, and mothers with overall or abdominal obesity had children with higher BAZ.

## Introduction

Extreme nutritional statuses, including undernutrition, stunting and obesity, are known to negatively affect women's reproductive functions [1], [2] and are associated with a plethora of maternal and child outcomes. Previous research had shown that maternal anthropometry, such as a high body mass index (BMI) and low height, was related to several adverse outcomes in the offspring, including the nutritional outcomes [3], [4], [5], [6].

Some lines of evidence can be found to either support or refute the relationship between maternal and offspring nutritional status, but most of them only use maternal height as a measure for maternal nutritional health. For instance, maternal height influences the linear growth of offspring over the growing period [6]; thus, the mother's height is positively related to the offspring's height, and stunting mothers are more prone to have stunting children [4], [5], [6]. Some studies regarding the effects of maternal adiposity (measured by BMI) on offspring outcomes have also been observed to be positively associated with children's BAZ [7] or with a greater weight-for-height index [8]. Gestational weight gain was associated with an increased BMI in childhood as well, which is extended through adulthood and includes an increased risk of obesity in adults [9]. This intergenerational transfer of malnutrition could be explained by the shared environmental and genetic factors [10], [11], [12]. Furthermore, it would be informative to investigate other nutritional relationships such as maternal BMI and central obesity with the resulting offspring height.

Although the rate of undernutrition has been decreasing worldwide while obesity rates increase, these extreme conditions might coexist in low- and middle-income countries such as Brazil, a country that is rapidly transitioning with an emerging economy [7], [13]. In this scenario, a careful assessment of the relationship between the nutritional statuses of mother-child pairs would be important to understand the complexity of the shifting patterns of nutritional transition in Brazil.

The objective of this study is to examine the association between maternal nutritional status and the occurrence of a nutritional impairment in their offspring, using a nationally representative sample of the Brazilian female reproductive-aged population and three maternal anthropometric measures: height, BMI and waist circumference (WC).

## Materials and Methods

### Dataset

We performed a secondary analysis using the publicly available data from the 2006 Brazilian Demographic Health Survey (DHS) [14], [15], which is a nationally representative cross-sectional household survey of women of reproductive age and children less than five years old in Brazil. DHS surveys have a high methodological quality and allow comparisons between countries.

The DHS sample has a probabilistic and complex design. In addition, the sample units were selected in two stages within each stratum: the census sectors, which were the primary sample units, and the households, the secondary sample units. Strata were defined by urban *versus* rural situation of the household and the Brazilian geographical regions: North, Northeast, Southeast, South, and Center-West, totaling ten sampling strata [14]. An eligible household had at least one woman resident of reproductive age (15 to 49 years old). Their pregnancies and any children born after 2001 until data collection were also targeted. The sample weight of participants was constructed through expansion and calibration methods. Thus, it accounted for the household, participants, number of eligible women per household, and non-response. Afterwards, calibration consisted of the computation of weights, considering the sex, the age group, the number of residents in each Brazilian region, and the number of households with at least one eligible woman. Calibration was based on the official population estimates from the Brazilian Institute of Geography Statistics [14].

The survey was carried out between November 2006 and May 2007 and included two structured questionnaires administered by trained personnel in a face-to-face interview; the survey included the household and women's characteristics, reproductive history, birth history of any offspring born after 2001, food security, breastfeeding, infant feeding, infant morbidity and sociodemographic characteristics. Anthropometric measurements were obtained from the mothers and children and followed standard procedures [16]. Two measurements were taken of each anthropometric indicator, and the mean value was calculated for each subject. Length/height and weight were measured using stadiometers with 1 mm of precision and an electronic scale with a 100 grams precision, respectively, and were calibrated every working day both before and after activities. Waist circumference was measured in a standing position, to the nearest millimeter, using a non-extendable measuring tape, at exactly halfway between the margin of the lowest rib and the iliac crest. These measurements were collected by trained personnel who were evaluated in the accuracy and precision of their measurements against a gold-standard anthropometrist. Detailed information regarding sampling plans, data collection information, data quality assurance and more is available in the DHS 2006 survey final reports at http://bvsms.saude.gov.br/bvs/pnds/index.php.

### Data Analyses

**Figure 1:**
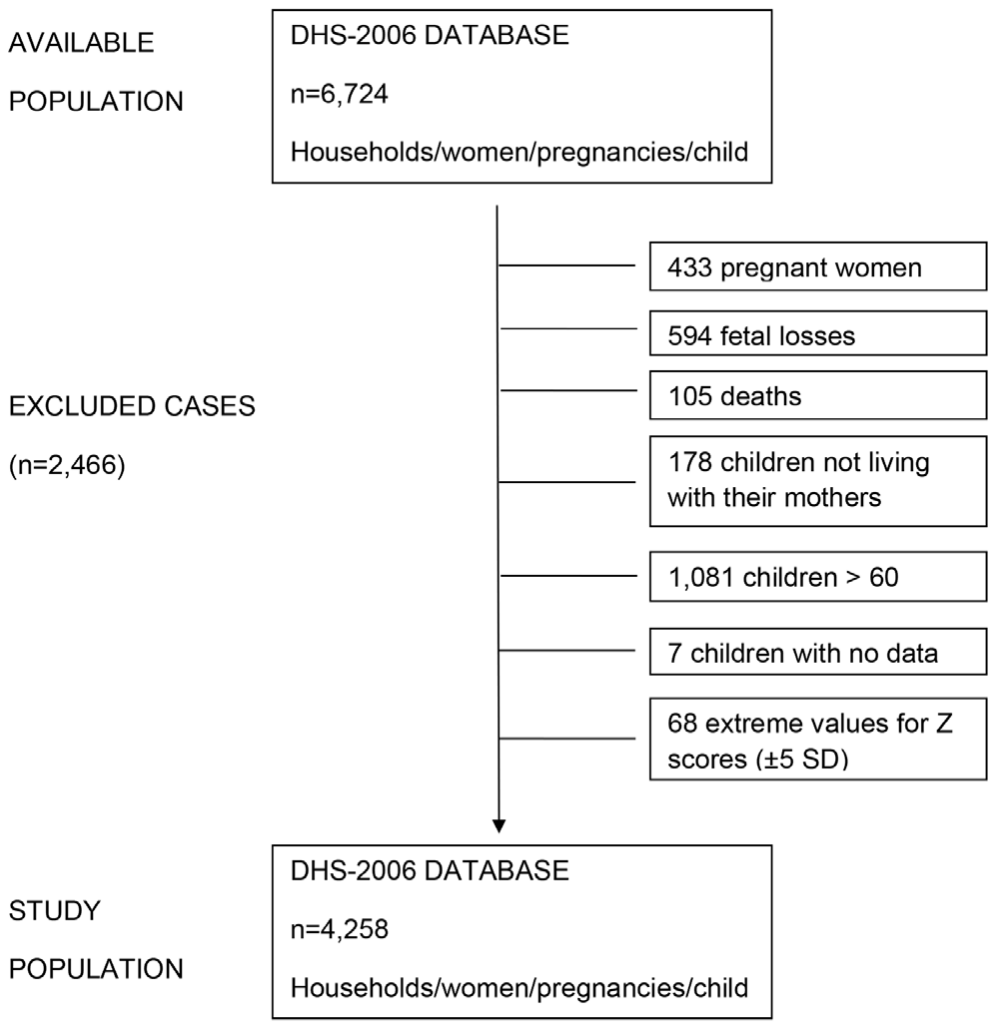
Study population diagram.

Each unit of observation in this dataset corresponds to the outcome of a singleton pregnancy, independent of a live birth or not. First, the available sample of 6,724 women was interviewed about their single pregnancies and children born between 2001 and the final data collection date. Of the 6,724 women and pregnancies examined, we excluded from the analysis those who were pregnant (n = 433) at the moment of interview; abortions/stillbirths (n = 594); child death before data collection (n = 105); children not living with their mothers (n = 178); children over 60 months of age (n = 1,081); extreme z-score values (±5 SD) (n = 68), which may be implausible; and children without any data (n = 7). The final dataset comprised 4,258 non-pregnant women with their children aged 0–60 months (**Figure 1**).

In this study, maternal anthropometry was the exposure of interest. Height, weight, BMI and waist circumference were the measurements used to describe the maternal nutritional status. Height was categorized in the following groups: <145 (stunting), 145–149, 150–154, 155–159, and ≥160 cm. Height and weight were used to calculate BMI according to the formula weight/height^2^ and were classified into the categories previously established by WHO [16], [17]: undernutrition (<18.5 kg/m^2^), eutrophic (18.5–24.9 kg/m^2^), overweight (25.0–29.9 kg/m^2^), obesity class I (30.0–34.9 kg/m^2^) and obesity class II–III (≥35.0 kg/m^2^). Waist circumference categories were also determined according to the international cut-off points [17]: normal (<80 cm), overweight (80–87.9 cm) and obesity (≥88 cm).

We investigated the relationship of maternal nutritional status with two nutritional indexes of children, height-for-age (HAZ) and body mass index-for age (BAZ) in z-scores, both of which are calculated using the WHO standard reference curves [18]. These indexes were used as continuous outcomes and were also categorized following the international conventions [16], [19]: stunting (HAZ<−2 SD) and overweight/obesity (BAZ>2 SD).

An unconditional subpopulation analysis was performed, restricting the estimation to subpopulations of interest; thus, we treated the exclusion conditions presented and missing values as the category 0 of the subpopulation indicators and the population of interest as category 1 [20]. This type of performance is highly recommended when analyzing complex, designed data because it is a more appropriate approach to the variance estimates [20]. We performed the analyses using the STATA software package, version 12.0 (Stata Corp., College Station, TX, USA) and its survey commands to account for the complex sample survey data composition: strata, clusters and weights.

First, we estimated the HAZ and BAZ averages (mean±SE) by maternal variables, including their nutritional status, and household characteristics. Overall differences across the categories were tested with the design-based Wald test. Next, we carried out a linear regression to estimate mean differences and 95% confidence intervals (95% CI) for each index by categories of maternal nutritional status indicators. Tests for linear trends were performed to examine whether there was a linear relationship between the categories of maternal nutritional status. The same analyses were repeated to obtain adjusted estimates, adjusting for maternal age, maternal education, smoking status, maternal parity, household income and household food security. The model for waist circumference was additionally adjusted for maternal height and maternal BMI. We used the Wald design-based test to evaluate the contribution of each variable in the model.

Based on theoretical model [13], the following variables were considered potentially confounding, as follows: quartiles of household income (in 2006–07, US $0.47 per *real*), maternal education in years (0, 1–4, 5–8, 9–11, 12 or more years), maternal age (15–19, 20–24, 25–29, 30–34, 35–39, ≥40), maternal smoking status (yes or no), parity (0–1, 2–3, ≥4 children) and household situation (urban, rural). Household food insecurity (HFI) was considered a confounder as well and was determined using the Brazilian Food Insecurity Scale (EBIA), as previously adapted and validated [21], [22]. The sum of the points using the 15 yes/no questions was calculated for each household, and each household was classified as food secure (0 points), mildly food insecure (1–5 points), moderately food insecure (6–10 points) or severely food insecure (11–15 points).

Additionally, we estimated prevalence ratios (PR) and 95% CI using Poisson regression models with robust standard errors for the categorical outcomes, stunting and overweight/obesity.

### Ethics

The DHS was conducted following the ethical standard pattern established by the Helsinki Declaration and approved by the Ethical Committee Council of the State Health Secretary of Sao Paulo. Data were de-identified, all the participants were informed of the research objectives and their rights, and signed the informed consent form. The DHS databases are public domain and can be accessed by anyone who is interested in secondary data analysis (http://bvsms.saude.gov.br/bvs/pnds/index.php).

## Results

The mean±SE age of the children was 29.1±0.5 months; 47.6% of the children were girls. The overall mean height-for-age and BMI-for-age z-scores were −0.31 and 0.43, respectively. The lower mean HAZ was observed among children of male sex and older ages and among children whose mothers presented a lower educational level and income and a higher parity and higher food insecurity level (p<0.05)(**Table 1**). We found no relationship between smoking, household situation and marital status with HAZ. Higher mean BAZ was observed among older children and children whose mothers had a higher educational level and income (p<0.05). BAZ averages did not differ across the categories of the other maternal and household characteristics.

**Table 1:** Mean height- and body mass index-for-age z-scores according to population characteristics, DHS/Brazil, 2006.

Child, maternal and household characteristics	n^1^	HAZ^2^	p^4^	n^1^	BAZ^5^	p^4^
		Mean (SE)^3^			Mean (SE)^3^	
**Child's sex**			0.04			0.41
Male	2,059	−0.37 (0.05)		2,048	0.46 (0.04)	
Female	1,949	−0.23 (0.05)		1,941	0.41(0.04)	
						
**Child's age (months)**			<0.0001			<0.0001
0–12	867	0.20 (0.09)		861	0.14 (0.07)	
13–24.99	766	−0.38 (0.10)		760	0.49 (0.08)	
25–36.99	780	−0.47 (0.06)		777	0.62 (0.05)	
37–48.99	810	−0.43 (0.06)		808	0.57 (0.06)	
49–60	785	−0.33 (0.08)		783	0.35 (0.06)	
						
**Child's HAZ**	—	—		3,989	0.45 (0.03)	0.04
						
**Child's BAZ**	4,008	−0.30 (0.04)	0.15	—	—	
						
**Maternal age (years)**			<0.0001			0.70
15–19	342	−0.45 (0.13)		337	0.37 (0.12)	
20–24	1,164	−0.44 (0.06)		1,162	0.40 (0.06)	
25–29	1,141	−0.19 (0.08)		1,134	0.42 (0.06)	
30–34	759	−0.13 (0.06)		756	0.52 (0.06)	
35–39	398	−0.33 (0.13)		398	0.49 (0.09)	
≥40	204	−0.34 (0.12)		202	0.50 (0.11)	
						
**Maternal education (years)**			<0.0001			0.03
0	117	−1.00 (0.28)		119	0.48 (0.20)	
1–4	906	−0.63 (0.07)		904	0.28 (0.07)	
5–8	1,447	−0.30 (0.05)		1,438	0.51 (0.05)	
	1,279	−0.11 (0.07)		1,272	0.40 (0.05)	
12+	237	−0.28 (0.16)		234	0.63 (0.11)	
						
**Maternal parity**			<0.0001			0.14
0–1	1,291	−0.20 (0.06)		1,281	0.46 (0.06)	
2–3	1,991	−0.31 (0.05)		1,982	0.44 (0.04)	
≥4	726	−0.70 (0.07)		726	0.31 (0.06)	
						
**Maternal marital status**			0.87			0.30
Married	3,406	−0.31 (0.05)		3,391	0.45 (0.03)	
Single	226	−0.33 (0.14)		225	0.23 (0.20)	
Widowed	19	−0.10 (0.30)		19	1.18 (0.44)	
Divorced	355	−0.25 (0.12)		352	0.44 (0.10)	
						
**Maternal smoking status**			0.06			0.91
No	3,402	−0.48 (0.11)		3,386	0.45 (0.11)	
Yes	606	−0.27 (0.04)		603	0.43 (0.03)	
						
**Household situation**			0.29			0.44
Urban	2,636	−0.28 (0.04)		2,624	0.44 (0.04)	
Rural	1,372	−0.41 (0.11)		1,365	0.39 (0.05)	
						
**Household income** 6 **(quartiles)**			<0.0001			0.001
1st Quartile (0–270)	866	−0.60 (0.07)		865	0.25 (0.06)	
2nd Quartile (271–500)	1,011	−0.42 (0.08)		1,006	0.40 (0.06)	
3rd Quartile (501–972)	771	−0.15 (0.06)		767	0.59 (0.07)	
4th Quartile (≥973)	908	−0.13 (0.08)		903	0.48 (0.07)	
						
**Household food security**			<0.0001			0.34
Food secure	1,926	−0.15 (0.06)		1,913	0.46 (0.04)	
Mild food insecurity	1,139	−0.38 (0.05)		1,113	0.44 (0.06)	
Moderate food insecurity	494	−0.54 (0.09)		493	0.36 (0.08)	
Severe food insecurity	327	−0.80 (0.11)		329	0.29 (0.11)	

Note: ^1^Sample crude n;

2 HAZ: height-to-age z-score;

3 Mean and standard error accounting for the complex survey design;

4 Overall F-test;

5 BAZ: body mass index-to-age z-score;

6 In 2006–07, 1 real  =  US$ 0.47.

We next examined the associations of maternal anthropometric indicators with the children's HAZ (**Table 2**). Maternal height and waist circumference, but not maternal BMI, were positively associated with the HAZ in a linear fashion. After adjustment for maternal age, educational level, parity, smoking status, household income and food security, children whose mothers' height was <145 cm had a 1.2 lower HAZ than those whose mothers were ≥160 cm tall (p for trend <0.0001). After further adjustment for maternal height and maternal BMI, the children of mothers with a waist circumference ≥88 cm had a 0.3 higher HAZ than those of mothers with a WC<80 cm (p for trend <0.01).

**Table 2:** Children height-for-age z-scores according to maternal anthropometry, DHS/Brazil, 2006.

Maternal anthropometry		HAZ					
	N	Mean (SE)	p^1^	Unadjusted difference (95%CI)	p^2^	Adjusted^3^ difference (95%CI)	p^2^
**Height**		3,995	<0.0001		<0.0001		<0.0001
<145 cm	123	−1.22 (0.14)		−1.29 (−1.59, −0.98)		−1.15 (−1.45, −0.85)	
145–149 cm	397	−0.99 (0.07)		−1.05 (−1.24, −0.87)		−0.94 (−1.13, −0.74)	
150–154 cm	964	−0.58 (0.06)		−0.64 (−0.80, −0.49)		−0.57 (−0.74, −0.40)	
155–159 cm	1,228	−0.27 (0.06)		−0.33 (−0.50, −0.17)		−0.25 (−0.42, −0.08)	
≥160 cm	1,283	0.07 (0.07)		Reference		Reference	
							
**Body Mass Index**		3,990	0.09		0.06		0.14
Undernutrition (<18.5 kg/m^2^)	174	−0.57 (0.18)		−0.21 (−0.56, 0.14)		−0.12 (−0.45, 0.22)	
Eutrophic (18.5–24.9 kg/m^2^)	2,173	−0.36 (0.04)		Reference		Reference	
Overweight (25–29.9 kg/m^2^)	1,067	−0.15 (0.08)		0.21 (0.04, 0.37)		0.16 (−0.01, 0.34)	
Obesity I (30–34.9 kg/m^2^)	399	−0.26 (0.11)		0.10 (−0.11, 0.31)		0.10 (−0.10, 0.30)	
Obesity II–III (≥35 kg/m^2^)	177	−0.40 (0.09)		−0.05 (−0.24, 0.15)		−0.07 (−0.30, 0.16)	
							
**Waist circumference**		3,901	<0.0001		<0.0001		0.01
Eutrophic (<80 cm)	1,836	−0.46 (0.05)		Reference		Reference	
Overweight (80–87.9 cm)	943	−0.20 (0.07)		0.26 (0.09, 0.42)		0.21 (0.04, 0.38)	
Obesity (≥88 cm)	1,122	−0.14 (0.07)		0.32 (0.15, 0.48)		0.32 (0.06, 0.58)	

Note: ^1^Overall F-test;

2 Test for linear trend;

3 mean differences adjusted for maternal age, maternal education, smoking status, maternal parity, household income and household food security.

The model for waist circumference was additionally adjusted for maternal height and maternal BMI. Sample sizes for adjusted models were 3,420, 3,415 and 3,335 for maternal height, BMI and WC, respectively. HAZ: height-to-age z-score; WC: waist circumference; BMI: body mass index.

We also examined the associations of maternal anthropometry with the prevalence of child stunting (HAZ<−2 SD) (**Table 3**). In multivariable analyses, maternal height was inversely related to prevalence of stunting, following a linear trend. Adjusted prevalence ratios and 95% CI for the categories of maternal height <145, 145–149, 150–154, 155–159 and ≥160 cm were, respectively: 2.95 (1.51–5.77), 2.29 (1.33–3.93), 1.09 (0.63–1.87), and 0.89 (0.45–1.77); (p for trend  = 0.001) (data not shown). In addition, the maternal WC, independent of the maternal BMI, height and socio-demographic characteristics, was inversely and linearly related to stunting (data not shown). Compared to mothers in the lowest quintile of WC (median = 69.3 cm), children whose mothers were in the highest quintile (median = 98.3 cm) had an 83% lower prevalence of stunting (p for trend = 0.001) (data not shown).

**Table 3:** Prevalence, unadjusted and adjusted prevalence ratios (PR) and 95% confidence intervals (95% CI) of stunted children according to maternal anthropometry, DHS/Brazil, 2006.

Women anthropometry	Stunting children – HAZ<−2 SD					
	% (SE)	p^1^	Unadjusted PR (95% CI)	P^2^	Adjusted^3^ PR (95% CI)	P^2^
**Height**		<0.0001		<0.0001		0.001
<145 cm	20.0 (4.4)		3.98 (2.10–7.55)		2.95 (1.51–5.77)	
145–149 cm	14.1 (2.3)		2.81 (1.58–4.98)		2.29 (1.33–3.93)	
150–154 cm	7.0 (1.2)		1.38 (0.83–2.30)		1.09 (0.63–1.87)	
155–159 cm	6.0 (1.4)		1.19 (0.60–2.36)		0.89 (0.45–1.77)	
≥160 cm	5.0 (1.2)		1.00		1.00	
						
**Body Mass Index**		0.23		0.32		0.57
Undernutrition (<18.5 kg/m^2^)	13.5 (6.5)		1.86 (0.69–4.97)		1.64 (0.63–4.26)	
Eutrophic (18.5–24.9 kg/m^2^)	7.3 (1.0)		1.00		1.00	
Overweight (25–29.9 kg/m^2^)	5.1 (1.0)		0.70 (0.44–1.12)		0.78 (0.48–1.27)	
Obesity I (30–34.9 kg/m^2^)	7.0 (2.0)		0.96 (0.54–1.71)		1.09 (0.63–1.89)	
Obesity II–III (≥35 kg/m^2^)	6.3 (2.6)		0.86 (0.37–2.00)		0.80 (0.29–2.22)	
						
**Waist circumference**		<0.0001		0.01		0.02
Eutrophic (<80 cm)	9.3 (1.4)		1.00		1.00	
Overweight (80–87.9 cm)	3.9 (0.7)		0.43 (0.28–0.66)		0.48 (0.27–0.84)	
Obesity (≥88 cm)	5.5 (1.0)		0.59 (0.39–0.89)		0.43 (0.19–0.95)	

Note: ^1^Design-based Pearson chi-squared test;

2 p for trend test;

3 Prevalence ratios adjusted for maternal age, maternal education, smoking status, maternal parity, household income and household food security.

The model for waist circumference was additionally adjusted for maternal height and maternal BMI. HAZ: height-to-age z-score; BMI: body mass index.

Finally, we examined the associations of maternal anthropometric indicators with the child's BMI-for-age z-score (**Table 4**). The BAZ was positively associated with maternal BMI and waist circumference in a linear manner but not with maternal height. After a multivariate adjustment, children whose mothers' BMI was ≥35 kg/m^2^ had a 0.4 higher BAZ than those whose mothers were eutrophic (18.5–24.9 kg/m^2^); (p for trend <0.0001). After further adjustment for maternal height and maternal BMI, waist circumference was no longer associated with the BAZ. Maternal anthropometry was not associated with the prevalence of child overweight/obesity (BAZ>2 SD) in this study (data not shown).

**Table 4:** Child body mass index-for-age z-scores according to maternal anthropometry, DHS/Brazil, 2006.

Women anthropometry		BAZ					
	n	Mean (SE)	p^1^	Unadjusted difference (95%CI)	p^2^	Adjusted^3^ difference (95%CI)	p^2^
**Height**			0.67		0.80		0.27
<145 cm	122	0.23 (0.20)		−0.19 (−0.60, 0.21)		−0.05 (−0.48, 0.37)	
145–149 cm	396	0.51 (0.08)		0.08 (−0.11, 0.28)		0.13 (−0.07, 0.33)	
150–154 cm	959	0.47 (0.05)		0.04 (−0.11, 0.18)		0.11 (−0.06, 0.27)	
155–159 cm	1,224	0.41 (0.06)		−0.01 (−0.17, 0.15)		0.01 (−0.17, 0.18)	
≥160 cm	1,276	0.43 (0.05)		Reference		Reference	
							
**Body Mass Index**			<0.0001		<0.0001		<0.0001
Undernutrition (<18.5 kg/m^2^)	171	−0.03 (0.12)		−0.40 (−0.64, −0.16)		−0.34 (−0.60, −0.08)	
Eutrophic (18.5–24.9 kg/m^2^)	2,164	0.37 (0.04)		Reference		Reference	
Overweight (25–29.9 kg/m^2^)	1,067	0.47 (0.06)		0.10 (−0.03, 0.23)		0.12 (−0.02, 0.26)	
Obesity I (30–34.9 kg/m^2^)	397	0.73 (0.08)		0.35 (0.18, 0.53)		0.37 (0.19, 0.55)	
Obesity II–III (≥35 kg/m^2^)	175	0.82 (0.12)		0.45 (0.19, 0.70)		0.42 (0.15, 0.69)	
							
**Waist circumference**			0.02		0.004		0.99
Eutrophic (<80 cm)	1,818	0.35 (0.04)		Reference		Reference	
Overweight (80–87.9 cm)	946	0.47 (0.05)		0.12 (−0.2, 0.25)		0.05 (−0.12, 0.23)	
Obesity (≥88 cm)	1,120	0.56 (0.06)		0.20 (0.06, 0.35)		−0.02 (−0.29, 0.25)	

Note: ^1^Overall F-test;

2 p for trend test;

3 mean differences adjusted for maternal age, maternal education, smoking status, maternal parity, household income and household food security.

The model for waist circumference was additionally adjusted for maternal height and maternal BMI. Sample sizes for adjusted models were 3,407, 3,404 and 3,325 for maternal height, BMI and WC, respectively. BAZ: body mass index-to-age z-score; WC: waist circumference; BMI: body mass index.

## Discussion

Using a large and representative nationwide sample of children and mothers from Brazil, we observed a strong positive association between maternal nutritional status and their offspring's nutritional status. More specifically, mothers of low stature had children with lower stature, and mothers with central obesity had taller children. These relationships were linear and independent of sociodemographic characteristics. We also found that mothers with overall or abdominal obesity had children with a higher BAZ compared to leaner women.

Thus, we found a relationship between maternal abdominal obesity and the children's HAZ, in which the children's HAZ increased as the maternal WC increased in a linear fashion. We also found a dose-response of this relationship when using the categories of maternal WC (WHO cut-off points and quintiles). Maybe these mother-child pairs are inserted in a household environment with excessive food availability contributing to an adequate linear growth; this environment could also contribute to previous maternal abdominal obesity or a longer period of time to attain a pre-pregnancy waist circumference measurement. Another possible and more speculative explanation could be the predisposition of diabetes in women with abdominal obesity [23], which may in turn create a intrauterine environment of insulin resistance and hyperglycemia, leading to fetal growth and childhood growth acceleration [10], [11], [24]. In addition to the faster, but not necessarily an excessive growth, these children are at an increased risk for obesity later in life [2], [11]. This particular finding signifies the need for further investigation on this matter.

Additionally, this study has shown a relationship between shorter mothers and offspring with a lower stature. This relationship is consistent with the findings from previous studies [4], [5], [6], [25], [26], is well established and has biological plausibility [27], [28]. The attained height of an adult is also a consequence of the nutritional environment in early life [29] and reflects the health stock accumulation through exposures of the environment [27]. For example, stunting women may provide a restricted nutrient uterine environment, leading to an inadequate supply for the fetus, which restricts fetal growth and promotes low birth weight and stunting in the offspring [1], [28]. This association also suggests an intergenerational transfer of the maternal poor health and socioeconomic adversity to the child [5], [12].

Another key finding of this study was that women with global or central obesity are more prone to having children with a higher BAZ, which corroborates results from previous investigations [7], [8], [9], including longitudinal ones [10]. This reinforces maternal BMI as being an important predictor of children's BMI as well as a major factor for the intergenerational transfer of body weight status. Although some caveats are involved in the mechanism of this relationship, maternal obesity may program the offspring's obesity, leading to chronic inflammation, oxidative stress and also insulin resistance and diabetes [2], [30], [31]. These conditions are related to placentation impairment, metabolism changes and changes in growth and body mass composition [2], [30], [32], which may affect the offspring throughout their lifetime [2], [9]. We found a weak association between maternal nutritional status and the offspring's BMI that could be explained by the prevalence of overweight and obesity in Brazil, which remains low among children aged 0–60 months. Moreover, it was previously suggested that the immediate effects of maternal obesity on children may only be increased upon exposure to an obesogenic environment later in life [2], [9].

We believe our findings are consistent for many reasons. First, DHS data are considered to be of high quality, and in most cases, they are the only source of maternal and child health data some countries have for surveillance, especially in the poor and transitional economies. Second, the standardized procedures used by these surveys increases the quality of the data and also allows for comparability across countries. Third, it provides a nationally representative sample, allowing for drawing conclusions about the entire country and from which we studied all the mother-child pairs available (0–60 months). Fourth, despite the fact that the DHS uses standardized procedures to be applied worldwide, this version of Brazilian DHS (2006) has introduced several additional items that are systematic and prospectively collected to be investigated in this representative sample of the children in the Brazilian population who are female and under five. Compared with other analyses derived from a common DHS, this additional information is a major advantage, allowing for new insights and inferences. In addition, we believe we used the appropriate statistical methods to investigate these relationships, controlling for important confounders, according to the theoretical frameworks.

With respect to other alternative explanations of reported associations, the cross-sectional design may constraint the temporality of a potential causal inference, but it is not possible to assume reverse causality between a child's anthropometric failure or a higher BAZ with the final maternal height, weight or waist circumference. Furthermore, we acknowledge the use of maternal anthropometric markers as surrogates of maternal nutritional health. These are good proxy for their nutritional status at the moment of pregnancy, independent of a possible weight gain or loss after the end of each pregnancy until the date of interview. Moreover, this is a more reliable assumption for height but not necessarily for BMI and WC. Although these data limitations exist, we observed a consistency of the reported associations across the models and sensitivity analyses.

In summary, our study provides evidence that maternal nutrition is highly and directed linked to their offspring's nutrition, although some mechanisms and pathways throughout this intergenerational transfer of nutritional health function remain unknown. Further investigation should continue the search for the biological explanations and plausibility. Our results corroborate the already-highlighted necessity of investments and public health policies focused on maternal and child care in Brazil, enabling early interventions and contributing to break this intergeneration cycle.
